# RNAkines Are Secreted Messengers Shaping Health and Disease

**DOI:** 10.1016/j.tem.2023.12.004

**Published:** 2023-12-29

**Authors:** Jing Li, Jingwen Fang, Xiaohong Jiang, Yujing Zhang, Antonio Vidal-Puig, Chen-Yu Zhang

**Affiliations:** 1 Nanjing Drum Tower Hospital Centre of Molecular Diagnostic and Therapy, State Key Laboratory of Pharmaceutical Biotechnology, Jiangsu Engineering Research Centre for MicroRNA Biology and Biotechnology, NJU Advanced Institute of Life Sciences (NAILS), Institute of Artificial Intelligence Biomedicine, School of Life Sciences, Nanjing University, Nanjing, Jiangsu 210023, China; 2 Wellcome-MRC Institute of Metabolic Science, Addenbrooke’s Hospital, University of Cambridge Metabolic Research Laboratories, Cambridge, UK; 3 Cambridge University Nanjing Centre of Technology and Innovation, Nanjing, China; 4 Research Unit of Extracellular RNA, Chinese Academy of Medical Sciences, Nanjing, Jiangsu 210023, China; 5 Plastic Surgery Hospital, Chinese Academy of Medical Sciences and Peking Union Medical College. Beijing, 100144, PR China

**Keywords:** Extracellular noncoding RNA, RNAkine, Metabolism, Cancer

## Abstract

Extracellular noncoding RNAs (ncRNAs) play crucial roles in intercellular communications. The process of ncRNA secretion is highly regulated, with specific ncRNA profiles produced under different physiological and pathological circumstances. These ncRNAs are transported primarily via extracellular vesicles (EVs) from their origin cells to target cells, utilising both endocrine and paracrine pathways. The intercellular impacts of extracellular ncRNAs are essential for maintaining homeostasis and the pathogenesis of various diseases. Given the unique aspects of extracellular ncRNAs, we propose the term “RNAkine” to describe these recently-identified secreted factors. In this article, we explore the roles of RNAkines as intercellular modulators, particularly in their ability to regulate metabolism and influence tumorigenesis, highlighting their definition and importance as a distinct class of secreted factors.

## Noncoding RNAs Are Secreted Regulatory Factors in Intercellular Communications

Historically, it was assumed that RNAs functioned solely within the cells where they were produced. However, a paradigm shift has occurred in recent years, as emerging evidence demonstrates that noncoding RNAs (ncRNAs) also play regulatory roles in distant target cells, influencing specific regulatory networks. This shift began with a significant discovery in 2007 and 2008, when several research groups independently reported finding mRNAs and microRNAs (miRNAs) (see Glossary) in the extracellular space [1, 2]. Our team and other researchers conducted ground-breaking studies that confirmed the presence of circulating miRNAs and revealed their unique expression patterns in the bloodstream, which are associated with specific diseases [3–5]. These findings position circulating miRNAs as promising diagnostic and prognostic biomarkers (Box 1). Inspired by the discovery of circulating miRNAs, further studies have explored other types of ncRNAs in circulation, such as long noncoding RNAs (lncRNAs), circular RNAs (circRNAs), tRNA-derived small RNAs (tsRNAs), and small nucleolar RNA (snoRNAs) [6]. Like miRNAs, the expression patterns of these ncRNAs are indicative of physiological or pathological states, reinforcing their potential as diagnostic biomarkers [7, 8].

The discovery of **extracellular ncRNAs** has sparked broad interest in their functional roles. Notably, their circulation in the bloodstream is just one part of their journey. Valadi et al. pioneered the model that mRNAs and miRNAs travel through the extracellular space encapsulated within extracellular vesicles (EVs) released by cells. They found that these RNAs, facilitated by EVs, shuttle between cells, introducing an alternative mechanism of gene-based intercellular communication [2]. Furthermore, Zhang et al. demonstrated the selective packaging of various extracellular miRNAs into EVs. They provided ground-breaking evidence of the functional impacts of miRNAs in recipient cells by showing how monocyte-secreted miR-150 regulates the mRNA expression of c-Myb in recipient endothelial cells (ECs) [9]. EVs, consisting mainly of microvesicles and exosomes [10], play a crucial role in this process. They enable the stable transfer of diverse ncRNAs, ensuring their functionality within recipient cells far from their origin [2, 6, 9]. Moreover, extracellular ncRNAs can travel independently of EVs and are often associated with stabilising proteins, such as high-density lipoprotein (HDL) [11], or the RNA-binding protein (RBP) Argonaute2 (Ago2) complex [12]. As such, extracellular ncRNAs have emerged as alternative secreted factors of transmitting intercellular information, paralleling the roles of hormones and cytokines mediating intercellular communications.

Traditionally, inter-tissue and inter-organ communications have been attributed to signalling molecules, such as cytokines, chemokines, growth factors, and hormones, integral to orchestrating physiological responses and maintaining whole-body homeostasis. In a way, extracellular ncRNAs meet the functional criteria of these conventional secreted factors. First, the secretion of extracellular ncRNAs is a regulated process. It is evident from the finding that the compositions of extracellular ncRNAs differ substantially from their cellular counterparts [13]. Moreover, they are selectively secreted in response to specific stimuli, demonstrating a precise stimulus-secretion coupling mechanism (Box 2) [9, 14, 15]. Such regulated secretion enables ncRNAs to be released in a manner that corresponds with physiological or pathological states. Second, extracellular ncRNAs share similarities with conventional secreted factors in transmitting information between organs, tissues, and cells via paracrine, endocrine, or autocrine methods. However, unlike conventional signalling molecules that typically bind to receptors and initiate signalling pathways, most extracellular ncRNAs operate within recipient cells as endogenous RNAs. Encapsulated extracellular miRNAs, for instance, directly regulate the expression of various types of molecules, including transcription factors (TFs) [9, 16], functional enzymes [17], signalling receptors [18, 19] and associated molecules [14, 20], and growth factors [21, 22]. Additionally, encapsulated lncRNAs and circRNAs often function as a competitive endogenous RNAs (ceRNAs) for miRNAs [23, 24], modulating mRNA expression by “sponging” miRNAs, thereby affecting downstream pathways (often represented as lncRNA or circRNA /mRNA/pathway axes) [25]. Some ncRNAs can also act as ligands, triggering Toll-like receptors (TLRs). For example, liver-secreted miR-122, which circulates independently of EVs, binds to TLR-7 and activates the inflammatory response in alveolar macrophages [26]. Moreover, EV-delivered miR-29 and miR-21 from cancer cells can bind to TLR7 and TLR8 residing in endosomes of immune cells after internalisation [27]. These interactions illustrate that extracellular ncRNAs convey a complex language, signalling recipient cells about the homeostatic changes in origin cells.

Comprehensive studies have yielded valuable insights into the implications of extracellular ncRNA-mediated crosstalk in various physiological processes and diseases, as summarised herein. However, despite the growing recognition of unique functional characteristics and relevance of extracellular ncRNAs, there remains a lack of universally accepted terminology for unequivocally identifying these molecules, which adds complexity and uncertainty to their understanding. Given their distinctive attributes, we propose considering extracellular ncRNAs as a distinct category of functionally relevant secreted molecules. To better reflect their broad yet selective spectrum of activity and signalling property during intercellular communications, we introduce the term “RNAkine”. This unified and distinctive nomenclature highlights the efficiency of extracellular ncRNAs as regulators in a diverse range of essential biological and pathological processes.

In this article, we aim to provide an in-depth review of findings that offer valuable insights into the concept and significance of RNAkines, including miRNAs, circRNAs and lncRNAs. Our primary focus will be on elucidating their relevance to two crucial areas: metabolism and tumorigenesis. By diving into these specific contexts, we hope to emphasise the necessity and rationale behind introducing the term “RNAkine” as a distinct nomenclature for these secreted molecules.

## RNAkines Play Significant Roles in Contributing to Metabolism

**Figure 1:**
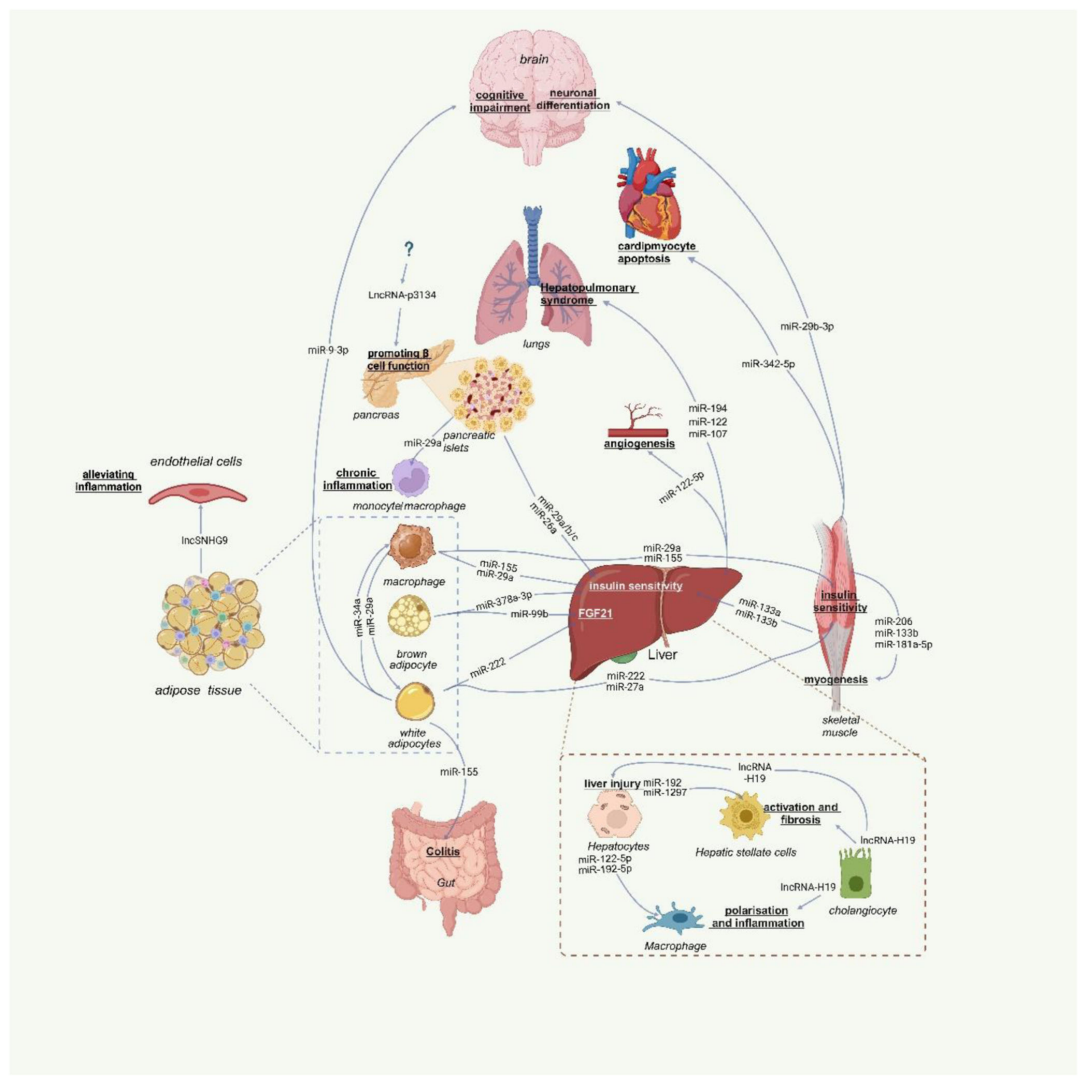
RNAkine-mediated metabolic regulatory network. The diagram illustrates the RNAkine-mediated metabolic regulatory network involving various secreted organs, including the pancreas, adipose tissue, skeletal muscle, and liver, which play critical roles in interorgan communication and metabolic regulation. The pancreas utilises RNAkines to regulate glucose homeostasis. Adipose tissue-secreted RNAkines are involved in regulating insulin sensitivity and glucose homeostasis. Skeletal muscle-secreted RNAkines contribute to the mechanisms underlying the metabolic benefits associated with physical activity. Liver-secreted RNAkines play pathological roles in various types of liver disease and pulmonary inflammation. This figure was generated using BioRender (https://biorender.com/).

RNAkines can function within recipient cells, even when originating from distant sources, suggesting their involvement in endocrine cell-to-cell and organ-to-organ communications. Several major organs, including the pancreas, adipose tissue, skeletal muscle, and liver, are integral components of metabolic networks. These organs rely on RNAkines to maintain interorgan coordination, which is critical in both metabolic homeostasis and disturbances (as illustrated in Figure 1 and summarised in Table 1).

### Pancreas-secreted RNAkines

The discovery of RNAkines secreted by pancreatic β cells in recent years has expanded our understanding of the regulation of glucose homeostasis, challenging the long-standing belief that insulin is the sole pancreatic β cell-derived hormone responsible for this function. This ground-breaking revelation, which occurred in 2018, just before the 100^th^ anniversary of the discovery of insulin, has revealed that β cells secrete increased RNAkines in response to stimuli such as K+, glucose, and arginine, which also trigger insulin secretion [28]. These stimuli induce the secretion of distinct patterns of RNAkines [28], indicating a tight control mechanism over RNAkine secretion induced by well-established insulinotropic triggers.

As speculated, RNAkines secreted from pancreatic β cells play significant roles in regulating glucose metabolism. Cellular miR-29 family members (miR-29a/b/c) can be considered “diabetes-related” miRNAs since increased miR-29 levels in adipose tissues [29] and skeletal muscle [30] promote insulin resistance. When there are high levels of free fatty acids (FFAs) in the bloodstream, which can result from physiological conditions, such as fasting, or pathological situations, such as a high-fat diet (HFD) or obesity, β cells release miR-29a/b/c into circulation in response to elevated FFA levels. These secreted miR-29 molecules are taken up by the liver, where they target p85α, promoting insulin resistance and disrupting systemic glucose homeostasis. Notably, genetic deletion of miR-29a/b/c in β cells reduces the circulating levels of miR-29 and improves HFD-induced hepatic insulin resistance [14]. Therefore, secreted miR-29 may act cooperatively with other obesity-associated factors to promote obesity-induced insulin resistance.

Furthermore, increased cellular miR-29a/b/c in β cells promotes chemotaxis, facilitating the recruitment of monocytes and macrophages [20]. These miR-29 molecules are also secreted and taken up by recruited macrophages, further promoting pro-inflammatory cytokine secretion [20]. As a result, both cellular and secreted miR-29 from β cells cooperate to trigger and promote inflammation in type 2 diabetes [20]. Additionally, miR-26a is another miRNA secreted by pancreatic β cells that improves hepatic insulin sensitivity [17]. Decreased levels of miR-26a in the blood of individuals with type 2 diabetes are associated with impaired insulin sensitivity [17]. These discoveries highlight the different roles of RNAkines in regulating glucose metabolism and the pathogenesis of insulin resistance in type 2 diabetes.

### Adipose tissue-secreted RNAkines

Adipose tissue is a multifaceted organ responsible for energy storage and the secretion of various bioactive adipokines, including hormones, signalling lipids, and inflammatory mediators [31]. Recent studies have highlighted the significant role of adipose tissue as a major source of circulating miRNAs since disrupting miRNA biogenesis in adipose tissues by deleting Dicer, a crucial protein for miRNA biogenesis, significantly reduces circulating miRNA levels [21].

Adipose tissue-secreted RNAkines play crucial roles in endocrine and paracrine communications with other organs, including the liver, skeletal muscle, colon and brain, modulating multiple biological processes. In obesity, the upregulation of secreted miR-27a disrupts glucose metabolism in skeletal muscle and leads to insulin resistance [32]. Additionally, miR-222 secreted from gonadal white adipose tissue (WAT) enters the skeletal muscle and liver, reducing insulin sensitivity by targeting insulin receptor substrate 1 (IRS1) [33].

The impacts of adipose tissue-secreted RNAkines extend beyond metabolic regulation. For instance, colitis can be exacerbated by miR-155 secreted from adipose tissues of mice fed a HFD [34]. Interestingly, HFD treatment shifts the profile of RNAkines from an anti-inflammatory to a pro-inflammatory phenotype, resulting in increased miR-155 expression [34]. Moreover, adipocyte-secreted EVs can traverse the blood-brain barrier (BBB) in a membrane protein-dependent manner and interact with neurons, particularly in the hippocampus [22]. In diabetes, adipose tissue-brain EV trafficking facilitates the transfer of miR-9-3p into the brain, causing synaptic damage and cognitive impairment by targeting brain-derived neurotrophic factor (BDNF) [22]. Therefore, adipocyte-secreted RNAkines provide a crucial link between diabetes and associated cognitive impairment [22].

Adipocyte-secreted RNAkines also play significant roles in regulating the biological function of cells within their local microenvironment. For example, adipocyte-secreted miR-34a inhibits the polarisation of M2 macrophage in adipose tissue, contributing to obesity-induced adipose inflammation by connecting lipid-overloaded adipocytes with inflammatory immune cells in obese adipose tissue [35].

Brown adipose tissue (BAT), a critical organ for thermogenesis, also functions as an endocrine organ, secreting conventional regulatory molecules, such as peptides and lipids, and RNAkines [36]. For example, BAT secretes miR-99b, which downregulates Fgf21 mRNA levels in the liver. The disruption of miR-99b secretion in mice with adipose tissue-specific Dicer knockout (AdicerKO) leads to upregulated Fgf21 expression in the liver [21], which can be reversed by administering exosomes loaded with overexpressed miR-99b in AdicerKO mice [21]. During cold exposure, activated BAT consumes substantial circulating glucose to fuel thermogenesis. In response to increased nutrient demand, the secretome of BAT plays a direct role in upregulating hepatic glucose output. For instance, IL-6 mediates hyperglycaemia under both acute (3 h) [37] and chronic (6 days) [38] cold exposure. BAT also secretes RNAkines, such as miR-378a-3p, in response to prolonged cold exposure, reprogramming systemic glucose metabolism by promoting hepatic gluconeogenesis [39]. This mechanism helps to maintain systemic glucose homeostasis, preventing hypoglycaemia when facing cold challenge [39]. It is also interesting to speculate how BAT-secreted RNAkines and other conventional secreted factors (such as IL-6) have co-dependent effects in response to different extents of cold exposure.

Macrophages within adipose tissue, or adipose-associated macrophages (ATMs), also secrete RNAkines that regulate insulin sensitivity. In obese mice, increased secretion of miR-155 from ATM impairs insulin sensitivity and glucose tolerance in insulin-target tissues, such as the skeletal muscle and liver [16]. Similarly, ATM-secreted miR-29a promotes insulin resistance in adipocytes, myocytes, and hepatocytes [40]. Conversely, anti-inflammatory M2-like macrophages secrete miR-690, which systemically improves insulin sensitivity [41].

### Skeletal muscle-secreted RNAkines

Skeletal muscle, traditionally known for its role in movement and energy storage, has emerged as a dynamic endocrine organ capable of secreting factors into circulation in response to various environmental and physiological challenges [42–44]. Recent research has unveiled the presence of functional RNAkines secreted by skeletal muscles. One group of miRNAs in muscles (known as myomiRs), including miR-1, miR-133a, miR-133b, miR-206, miR-208a, miR-208b, miR-486, and miR-499, are secreted into circulation via exosomes [45, 46]. Interestingly, the patterns of RNAkines secreted by muscles depend on the type of exercise performed. For instance, various exercises, such as downhill exercise [47], swimming training [46], and acute aerobic exercise [48], can result in different myomiR profiles in circulating exosomes. However, the mechanism underlying the selectivity of released RNAkines during different types of exercise is still unclear. Of note, the elevation in the levels of RNAkines is more likely to be observed immediately after exercise, regardless of the duration [49].

The secretion of RNAkines by muscle has garnered scientific interest due to their critical role in myogenesis. Muscle cells, particularly myoblasts and myotubes, communicate through these RNAkines, enhancing muscle cell development and differentiation. C2C12 myotubes, for instance, secrete RNAkines that regulate crucial signalling pathways, such as the Wnt signalling pathway, in myoblasts, facilitating muscle cell differentiation [50]. Exercise-induced RNAkines target genes in the MAPK pathway, thereby promoting muscle cell differentiation and growth [51]. Additionally, acute aerobic exercise-induced RNAkines, such as miR-206, miR-133b, and miR-181a-5p, have shown promise in preventing muscle dystrophy [52].

Moreover, RNAkines secreted by skeletal muscles during physical activity positively impact metabolic health. High-intensity interval training (HIIT), for example, induces the release of miR-133a and miR-133b from muscles, which improves hepatic insulin sensitivity by targeting the transcription factor Forkhead box O1 (FoxO1) in the liver [53]. Administration of exosomes loaded with miR-133a and miR-133b to mice enhances insulin sensitivity, highlighting the systemic metabolic influence of muscle-secreted RNAkines [53]. Additionally, long-term swimming training results in elevated levels of miR-342-5p in circulating exosomes, which inhibits cardiomyocyte apoptosis under hypoxia/reoxygenation conditions [46].

Furthermore, muscle-secreted RNAkines play a role in establishing communication between muscles and the brain. For instance, miR-29b-3p, upregulated in muscle cells in various types of atrophy, has been found to disrupt neuronal differentiation [54]. Ageing mice exhibit elevated levels of miR-29b-3p in both muscle and blood and *in vitro* studies have shown that atrophic muscle cells secrete miR-29b-3p, which downregulates neuronal-related genes and inhibits neuronal differentiation when taken up by neuronal SH-SY5Y cells [54]. Additionally, muscle-secreted RNAkines impact the function of ECs. For example, C2C12 myotube-secreted miR-130a promotes EC proliferation, migration, and tube formation by activating the nuclear factor-kappa B (NF-κB) pathway [55], suggesting an angiogenic role of muscle-secreted RNAkines and their involvement in skeletal muscle-mediated capillarisation.

### Liver-secreted RNAkines

The liver, a vital organ with numerous functions, including metabolism, detoxification, digestion, synthesis, and storage, also releases RNAkines under various pathophysiological conditions. In liver disorders characterised by fibrosis, RNAkines mediate communication between hepatocytes and hepatic stellate cells (HSCs), a key player in liver fibrogenesis. Hepatocytes release RNAkines that can induce the activation of HSCs. For instance, in response to palmitic acid (PA) treatment, hepatocytes increase exosome production and modify miRNA profiles in exosomes. This leads to elevated expression of miR-192, which stimulates the expression of fibrotic genes in HSCs [56]. Additionally, PA treatment induces the secretion of miR-107 from hepatocytes, triggering HSC activation by targeting DKK1 and activating Wnt signalling [57].

RNAkines released by hepatocytes also contribute to metabolic-associated fatty liver disease (MAFLD) pathogenesis. In response to lipotoxicity, hepatocytes release miR-1297, which promotes HSC activation and proliferation by modulating the PTEN/PI3K/AKT signalling pathway [58]. Inflammation is closely linked to the development of liver disease, with RNAkines mediating communication between hepatocytes and immune cells. Cholesterol, a significant contributor to MAFLD, induces the increased release of miR-122-5p from hepatocytes, which leads to M1 polarisation of macrophages and inflammation [59]. Lipotoxic injury results in the release of miR-192-5p, which activates macrophages and contributes to lipotoxicity-induced MAFLD by regulating the Rictor/Akt/Foxo1 pathway [60]. In alcoholic hepatitis (AH), hepatocyte-secreted miR-122 sensitises monocytes to LPS by targeting the HO-1 pathway, which exacerbates inflammation [61]. Inhibiting miR-122 may hold therapeutic potential for treating inflammation-related liver diseases [61]. Finally, hepatocyte-secreted miR-107 is delivered to CD4+ lymphocytes, which enhances the expression of IL-9 by modulating Foxp1[57].

Furthermore, liver-secreted RNAkines can impact distant organs. In hepatopulmonary syndrome (HPS), characterised by pulmonary vascular abnormalities, oedema, and dyspnoea, liver-secreted RNAkines contribute to its pathogenesis. Hepatocyte-secreted miR-194 enhances pulmonary angiogenesis, a critical process in HPS development [62]. Liver injury induces pulmonary inflammation through miR-122 secretion [26]. Interestingly, circulating miR-122, independent of EV transport, increases in individuals with liver damage [26]. Alveolar macrophages take up circulating miR-122, which triggers inflammatory responses by directly binding to TLRs [26]. Depleting miR-122 in mouse livers abolishes pulmonary inflammation and tissue damage [26]. However, liver-secreted RNAkines can also have beneficial roles. For example, exercise induces the release of miR-122-5p from the liver and enhances angiogenesis and wound healing [63]. However, this enhancement of angiogenesis may be a concern in the context of anticancer treatment strategies.

### Other types of RNAkines contributing to metabolic regulation and disease

Compared to those of miRNAkines, investigations of other types of RNAkines secreted by significant organs are still emerging. For instance, lncRNA-p3134, elevated in the serum of individuals with type 2 diabetes, plays a beneficial role in glucose homeostasis by promoting β cell function [64]. Adipocyte-secreted lncSNHG9 has protective properties, such as its roles in alleviating inflammation and apoptosis in ECs, which are achieved by suppressing the expression of TNF receptor type 1-associated death domain (TRADD) [65]. Interestingly, the levels of secreted lncSNHG9 are reduced in obese individuals with endothelial dysfunction, highlighting its role in preserving vascular physiology [65]. In a Drosophila study, adipose-derived circ_sxc maintains normal brain neuronal synaptic signalling [66]. The liver also contributes to lncRNAkine secretion, with cholangiocyte-enriched lncRNA-H19 being elevated in serum exosomes from a liver injury mouse model and individuals with cirrhosis. This lncRNA can be transferred to hepatocytes, suppressing the small heterodimer partner (SHP) at both transcriptional and posttranscriptional levels, thereby promoting cholestatic injury [67]. Furthermore, cholangiocyte-secreted lncRNA-H19 promotes fibrosis by activating HSCs [68]. Under cholestatic conditions, cholangiocyte-derived lncRNA-H19 can also activate M1 polarisation of Kupffer cells [69]. These findings collectively demonstrate the multifaceted regulatory functions of cholangiocyte-secreted lncRNA-H19 in liver diseases. In another context, the liver-secreted lncRNA MT1DP (metallothionein 1D pseudogene) can reach distant kidneys, where it reinforces the nephrotoxic effects of cadmium exposure [70].

## Cancer Cell-Derived RNAkines as Modulators of Tumour Microenvironment

**Figure 2:**
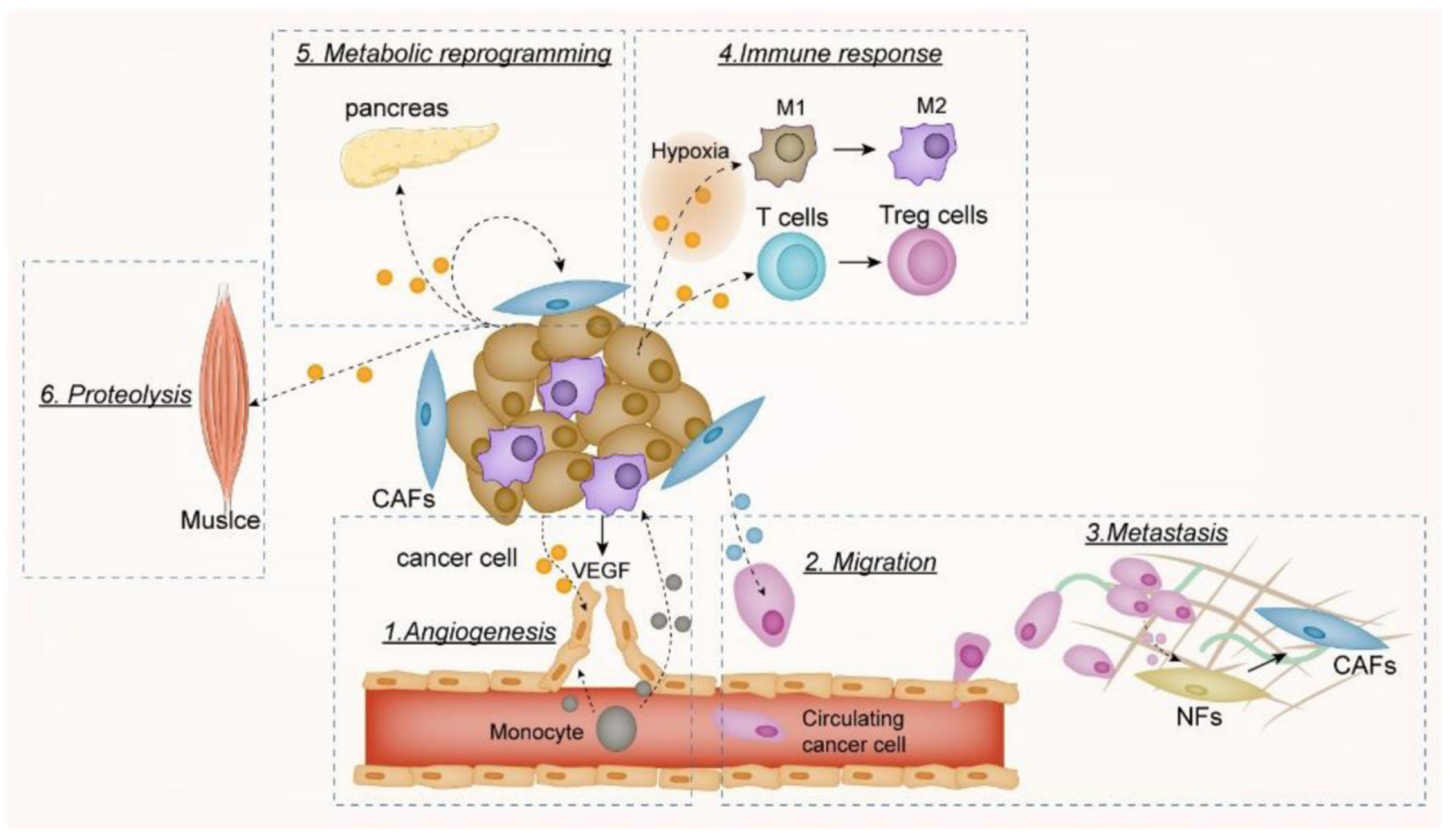
RNAkine-mediated crosstalk between malignant cancer cells and nonmalignant cells within the TME. Cancer cell-secreted RNAkines play crucial roles in various processes of tumorigenesis, including angiogenesis, migration, metastasis, immune envision and metabolic reprogramming. 1) RNAkines regulate EC migration, promoting angiogenesis; 2-3) RNAkines facilitate the formation of a metastatic niche in distant tissues. CAFs can also secrete RNAkines, contributing to the migration and progression of tumour cells, 4) RNAkines induce immune evasion by expanding Tregs and M2 polarisation; 5-6) RNAkines initiate metabolic reprogramming in both adjacent cells and distant organs. This figure highlights the complex RNAkine-mediated interplay between cancer cells and cells within the TME.

Tumorigenesis extends beyond the proliferation of cancer cells and involves multiple alterations within the tumour microenvironment (TME), which comprises ECs, cancer-associated fibroblasts (CAFs), and immune cells [71]. RNAkines, encompassing miRNAs, circRNAs, and lncRNAs, have emerged as modulators in the reciprocal interactions between cancer cells and nonmalignant host cells within the TME. These RNAkines govern critical processes, including angiogenesis, immune evasion, metastasis, and metabolic reprogramming [72]. Beyond their local effects, cancer cell-secreted RNAkines can also impact distant organs, such as skeletal muscle [73] and pancreas [74], orchestrating systemic metabolic adaptations to support tumour growth (as illustrated in Figure 2 and summarised in Table 2).

Evidence suggests that cancer cell-secreted RNAkines regulate EC function to favour angiogenesis across various cancers. Factors associated with tumorigenesis, such as hypoxia [75], tissue inhibitor of metalloproteinases-1 (TIMP-1) [76] and MYC [77, 78], can modulate the release of proangiogenic RNAkines. These RNAkines can enhance angiogenesis by upregulating proangiogenic factors. For example, colorectal cancer (CRC)-derived miR-25-3p upregulates vascular endothelial growth factor receptor 2 (VEGFR2), ZO-1, occludin, and claudin 5 by targeting KLF2 and KLF4 [79]. LncRNA-H19 from CRC competes with miR-138 for its target, the HIF-1α, activating the HIF-1α/VEGF axis. Additionally, ovarian cancer cell-secreted miR-205 augments angiogenesis by regulating the PTEN-AKT pathway [80]. It is not only cancer cells that contribute to angiogenesis by releasing RNAkines. Monocytes secrete miR-150 to enhance EC migration, tube formation, and angiogenesis [9, 81] by triggering VEGF secretion [82].

Tumour metastasis is a formidable challenge in cancer therapy and is often a leading cause of mortality. Cancer cells release RNAkines to create a conducive environment for metastasis [83]. Cancer cells with low metastatic potential can acquire an enhanced metastatic capacity by assimilating RNAkines released from highly metastatic cancer cells [84]. Highly metastatic hepatocellular carcinoma (HCC) cells secrete miR-1247-3p, which transforms normal fibroblasts into CAFs by activating the β1-integrin-NF-κB signalling pathway, creating a supportive niche for tumour metastasis [85]. Cancer cell-secreted RNAkines also contribute to metastasis by regulating the epithelial-mesenchymal-transition (EMT) process, exemplified by miR-92a-3p released from highly metastatic cancer cells [84]. Furthermore, RNAkines mediate the crosstalk between cancer cells and ECs to facilitate metastasis. LNMAT2, a lncRNA selectively loaded into exosomes via its interaction with hnRNPA2B1, can be internalised by human lymphatic endothelial cells (HLECs), epigenetically upregulating prospero homeobox 1 (PROX1) expression to enhance lymphatic metastasis [86]. Another example involves circRNA-100,338 secreted from HCC, which regulates the EC function and angiogenesis, ultimately enhancing metastasis [87].

Cancer cells employ various strategies to manipulate the local immune response within the TME, and RNAkines play crucial roles in these processes. A characteristic feature of tumour immune evasion is the expanded population of CD4+CD25highFoxp3+ regulatory T cells (Tregs). MiR-214, ubiquitously secreted by various types of human cancers and mouse tumour models, promotes Treg expansion by suppressing phosphatase and tensin homologue (PTEN) in recipient T cells [88]. Similarly, circGSE1 derived from HCC induces Treg expansion by regulating the miR-324-5p/TGFBR1/Smad3 axis, leading to a reduced immune response [89]. RNAkines also impact immune checkpoint molecules. Endoplasmic reticulum (ER) stress in HCC cells triggers the secretion of miR-23a-3p, which upregulates the expression of programmed death-ligand 1 (PD-L1) in macrophages [90]. Tumour-derived circRNA-002178 promotes the expression of programmed cell death protein 1 (PD-1) by sponging miR-28-5p in T cells, inducing immune evasion [91]. Cancer cell-secreted RNAkines govern tumour-associated-macrophage (TAM) polarisation (M2) within the TME by regulating PTEN. These RNAkines include miR-25-3p, miR-425-5p, and miR-130-3p [92], and their upregulation can be triggered by activating the CXCL12/CXCR4 axis in CRC cells [92]. M2 polarisation significantly contributes to tumour progression, aiding in tumour initiation, angiogenesis, and metastasis [93]. Hypoxia also plays a role in M2 polarisation through cancer cell-secreted RNAkines, including miR-21-3p [94], miR-125b-5p [94], miR-181d-5p [94], miR-301a-3p [95] and circ0048117 [96].

Metabolic reprogramming is another hallmark of cancer, with RNAkines participating in metabolic adaptation within the TME and distant organs. For instance, breast cancer (BC) cell-secreted miR-122 inhibits the glycolytic enzyme pyruvate kinase, suppressing glucose uptake by non-tumour cells in the pre-metastatic niche [97]. BC cell-secreted miR-105 reprograms the glucose and glutamine metabolism of CAFs, fuelling neighbouring cancer cells [98]. Interestingly, reprogrammed CAFs can convert metabolic waste into energy-rich metabolites [98]. BC cell-secreted miR-122 also has systemic effects on metabolism via its induction of proteolysis in skeletal muscle [73] and disruption of insulin secretion from the pancreas [74]. These insights partially elucidate the mechanisms underlying metabolic syndrome during cancer. RNAkines secreted from nonmalignant cells within the TME also promote glucose reprogramming in cancer cells. For example, TAM-secreted lncRNAs can regulate aerobic glycolysis in BC cells by stabilising HIF-1α [99].

The current evidence suggests that RNAkines form a complex intercellular signalling network. Broadly speaking, RNAkines operate in concert with conventional secreted factors, potentially regulating biological processes to varying degrees. Conventional secreted factors often work for the central control of the whole-body homeostasis. For example, leptin adjusts energy stores in response to overall energy balance of the body [100]. During tumorigenesis, host-derived cytokines can systematically regulate the immune system to suppress tumour formation [101]. In contrast, RNAkines typically work for the functionality of local tissues where they are produced or taken up. Examples include pancreatic islet-secreted RNAkine that regulates insulin sensitivity [17], BAT-secreted RNAkine that recruits fuel for thermogenesis [39], and cancer cell-derived RNAkines that promote tumorigenesis. This suggests a hierarchical interplay between RNAkines and conventional secreted factors within organisms. More efforts are necessary to fully elucidate the nuanced and distinct roles by conventional secreted factors and RNAkines.

## Challenges in RNAkine Research

Research into RNAkines has revealed their signalling properties, but scientists continue to face significant challenges. First, there is a lack of comprehensive documentation regarding the concentrations of RNAkines in circulation and in recipient cells, which adds uncertainty to their causative effects in recipient cells. However, it is noteworthy that some RNAkines have been reported to reach concentrations in the picomolar range (10^-12^ M) in the bloodstream, comparable to those of cytokines. For example, miR-16, a widely expressed RNAkine, maintains an approximate concentration of 2.6 pM in the human bloodstream [102]. miR-378a-3p, which is significantly increased in response to cold exposure, can reach a concentration of 0.1 nM in the bloodstream [39]. The concentration of RNAkines in recipient cells is also a significant concern. Some studies offer exact functional concentrations of RNAkines in recipient cells. For example, miR-378a-3p is increased from ∼102 fmol/g liver tissue to ∼182 fmol/g liver tissue upon cold exposure [39]. When converted to copies per liver cell, the miR-378a-3p level reaches approximately 300 copies per liver cell (157 million cells per g of mouse liver [103]), reaching the reported functional miRNA levels (> 100 copies per cell [104]). In the future, establishing a common standard for assessing RNAkines in circulation is necessary to address this challenge.

Second, the mechanisms controlling stimuli-induced RNAkine secretion remain largely unknown. Various stimuli can induce the secretion of RNAkine via different mechanisms, including 1) regulating RNAkine transcription by targeting specific TFs [77, 78, 98], 2) modulating the secretory machinery involved in EV biogenesis, such as ceramide [105], and 3) altering the expression of specific RBPs that sort RNAkines into the extracellular space, such as hnRNPA2B1 [106]. A deeper understanding of these mechanisms can enhance our appreciation of the physiological and pathological significance of RNAkines.

## Why Is New Terminology Needed for RNAs Outside the Cell?

The introduction of a new term, namely “RNAkine”, to describe a diverse class of ncRNAs actively secreted by cells in response to homeostatic changes and their role as mediators in intercellular communications, homeostasis and pathological processes addresses a critical need within the field of RNA research. Alternative terms, such as “**EV-RNAs**/**exosomal RNAs”** [107], highlighting their spatial localisation, have been proposed,. The term “secreted RNAs” refers to RNA that functions exclusively outside the cells that produce them [9]. While these terms have their merits, they fall short of capturing the unique characteristics of extracellular ncRNA. These characteristics include their selective release and potent signalling properties, which are essential to be considered bona fide factors of intercellular communications. Additionally, the field anticipates the discovery of RNAkines with other associations or biological sources, such as exogenous ncRNAs produced by other species, that may not fit neatly into existing categories.

The rationale behind coining the term “RNAkine” lies in the shared biological roles with conventional secreted factors known for their significant signalling capabilities. As noted above, RNAkines are distinct in their selective production and secretion in response to specific stimuli. They are crucial in conveying information between cells and organs, initiating cellular responses in recipient cells, and impacting gene expression, cell growth, immune responses, and metabolism. The suffix “-kine”, originating from the Greek word for “movement” or “motion”, aptly reflects the ability of the ncRNA to act at a distance. Thus, “RNAkine” effectively leverages this suffix to denote this unique characteristic.

**Figure 3:**
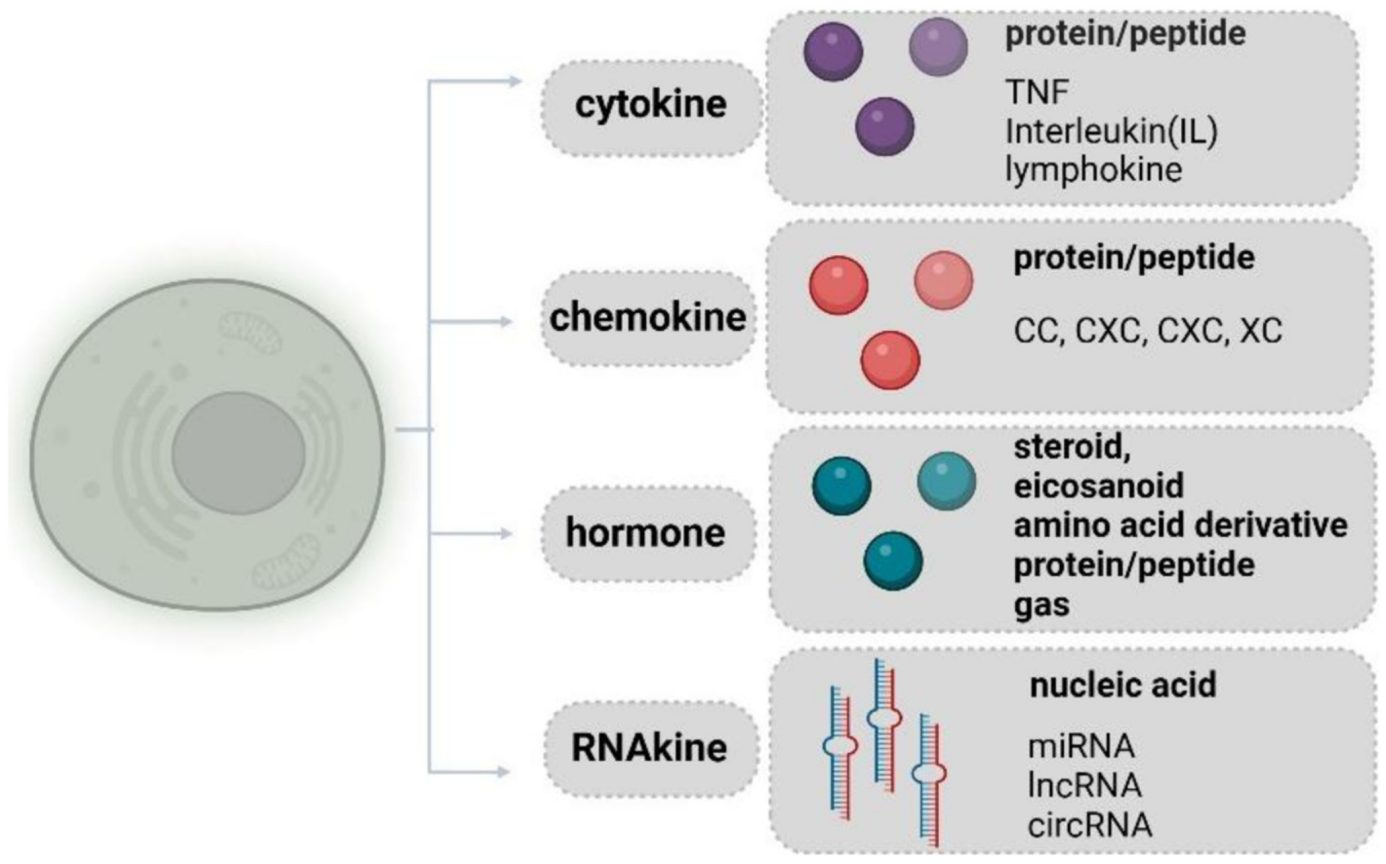
Classifications of secreted factors. In addition to conventional secreted factors, cells also secrete ncRNA molecules (RNAkines). The molecular types of secreted factors are presented in bold, and representative examples or subfamilies, such as chemokines, are listed. This figure provides an overview of the diverse types of secreted factors contributing to intercellular signalling. This figure was created using BioRender (https://biorender.com/)

More importantly, the term “RNAkine” helps differentiate functional extracellular ncRNAs from conventional secreted factors in several ways. First, the fact that extracellular ncRNAs are nucleic acids represents a significant qualitative distinction from cytokines or hormones, justifying the need for specialised nomenclature. Second, “RNAkine” utilises an alternative method for transferring information. They can simultaneously target different components of biological networks, leading to coordinated and integrated responses similar to the robust synergy seen in hormonal secretory patterns within complex biological processes. Third, “RNAkine” emphasises their different but co-dependent roles with conventional secreted factors in contributing to specific physiological processes or diseases. Finally, the term “RNAkine” encompasses various types of RNA molecules, creating a self-contained category that distinguishes them from non-functional extracellular RNAs or intracellular RNAs. (Figure 3). Thus, “RNAkine” provides a concise, comprehensive and precise representation of this emerging class of extracellular ncRNAs. This nomenclature fosters consensus and holds the potential for widespread acceptance within the scientific and clinical communities. Consequently, it assists scientists and clinicians in effectively exploring and discussing the roles played by these recently identified secreted factors in various critical biological processes.

## Concluding Remarks and Future Perspectives

In summary, exploring RNAkines has unveiled their previously unrecognised roles in various biological processes, from metabolic homeostasis, exercise-mediated benefits, insulin resistance, and tumorigenesis. These discoveries highlight the potential of RNAkines as promising therapeutic targets for many diseases due to their substantial involvement in these critical biological pathways.

Despite substantial progress in comprehending RNAkines, numerous challenges and avenues for future research remain (see Outstanding Questions). It is imperative to pinpoint organ- and cell-specific RNAkines and unravel the intricate mechanisms governing their selective packaging, secretion and functional roles. While evidence suggests that the uptake of RNAkines by recipient organs or cells is a selective process, the mechanisms underpinning this phenomenon, particularly in cases where miRNAs function endocrinologically, remain predominantly uncharted. Consequently, we eagerly await further studies and well-designed investigations to illuminate the aetiopathogenic mechanism mediated by RNAkines. The field of RNAkine research harbours immense potential for advancing our comprehension of intercellular communication and its repercussions on various physiological and pathological processes.

## Figures and Tables

**Table 1:** Functional RNAkines mediating crosstalk in metabolism.

RNAkine	Donor	Recipient	Target	Effect	Mechanism	Ref.
miR-29a/b/c	Pancreatic β cell	Liver	p85α	Impairing insulin sensitivity	Inhibiting PI3K/AKT signalling pathway	[14]
miR-26	Pancreatic β cell	Liver, VAT, BAT	ACSL3, ACSL4, TCF7L2, PCK1, GSKβ, PKCδ, PKC θ	Promoting insulin secretion and β cell replication (autocrine); improving peripheral insulin sensitivity (endocrine)	Activating insulin signalling pathway, inhibiting gluconeogenesis and lipid synthesis.	[17]
MiR-29	Pancreatic β cell	Monocyte/macrophage	TRAF3	Inducing inflammation in T2DM	Increasing chemotaxis of Monocytes and macrophages	[20]
miR-99b	BAT	Liver	Fgf21	Affecting insulin tolerance	Down-regulating *Fgf21* mRNA and circulating FGF21	[21]
miR-378a-3p	BAT	Liver	p110α	Improving glucose output during cold exposure.	Inhibiting PI3K/AKT signalling pathway	[39]
miR-27	WAT	Skeletal muscle	PPARγ	Impairing insulin sensitivity	Impairing insulin signalling pathway Glut 4 expression	[32]
miR-222	WAT	Liver, Skeletal muscle	IRS1	Impairing insulin sensitivity	Impairing insulin signalling pathway	[33]
miR-155	WAT	Macrophages in intestine *lamina propria*	--	Promoting DSS-induced colitis	Promoting M1 differentiation.	[34]
miR-9-3p	WAT	Neuron	BDNF	Damaging synapse and cognitive	Impairing formation, maintenance, and plasticity of synapse	[22]
miR-155	ATM	Skeletal muscle, Liver	PPARγ	Impairing insulin sensitivity	Impairing insulin signalling pathway	[16]
miR-29a	ATM	Adipocyte, Myocyte, Hepatocyte	PPARδ	Impairing insulin sensitivity	Impairing insulin signalling pathway	[40]
miR-690	M2	Adipose tissue, Liver, Skeletal muscle	NADK	Improving insulin sensitivity	Improving insulin responses	[41]
miR-34a	Adipose tissue	Macrophage in adipose tissue	KLF4	Promoting obesity-induced adipose inflammation	Inhibiting M2 polarization	[34]
Circ_sxc *(in Drosophila)*	Adipose tissue	Brain	Dme-miR-87-3p	Ensuring brain function	Sponging miR-87-3p and in turn regulating neurological receptor ligand proteins	[66]
LncSNHG9	Adipose tissue	EC	TRADD	Preventing EC dysfunction.	Inhibiting inflammation and apoptosis	[65]
miR-22, miR-181a, miR-133a	Myotubes	Myoblasts	SIRT1	Promoting myogenesis	Contributing to the commitment of myoblasts in the process of differentiation.	[50]
miR-133a, miR-133b	Skeletal muscle	Liver	--	Improving insulin sensitivity	Inhibiting gluconeogenesis through upregulating FoxO1	[53]
miR-29b-3p	Myotubes (Atrophy)	Neuron cells	c-FOS, BCL-2, RIT1, and LAMC1	Inhibiting neuronal differentiation	Activating c-FOS/HIF1α-AS2 pathway and neuronal differentiation-related genes.	[54]
miR-130a	Myotubes	ECs	--	Improving the proliferation, migration and tube formation of ECs	Activating NF-κB	[55]
miR-192	Hepatocytes	HSCs	--	Promoting HSC activation	Enhancing various of fibrosis-associated markers such as α-SMA, TGF-β, Col1a1.	[56]
miR-107	Hepatocytes	HSCs CD4+ lymphocyte	DKK1 Foxp1	Promoting HSC activation	Activating HSCs by activating Wnt signalling, and increasing the expression of IL-9	[57]
miR-1297	Hepatocytes	HSCs	PTEN	Promoting HSC activation	Contributing to the activation and proliferation of HSCs via regulating PTEN/PI3K/AKT signalling pathway	[58]
miR-122-5p	Hepatocytes	Monocyte	--	Inducing inflammation	Promoting the M1 polarisation	[59]
miR-192-5p	Hepatocytes	Macrophage	Rictor	Inducing inflammation	Activating M1 by modulating Rictor/Akt/FoxO1 signalling and promoting NAFLD progression	[60]
miR-122	Hepatocytes	Monocyte	HO-1	Inducing inflammation	Sensitising monocytes to LPS stimulation and increased levels of pro-inflammatory cytokines.	[61]
miR-122 (EV-independent)	Hepatocytes	Alveolar macrophage	--	Inducing inflammation in HPS	Activating inflammatory responses trough binding to TLR.	[26]
miR-122-5p	Hepatocytes	Endothelial	AGPAT1	Promoting angiogenesis	Enhancing fatty acid utilisation.	[63]
LncRNA-H19	Cholangiocytes	Hepatocytes	SHP	Promoting cholestatic liver injury	Supressing SHP expression	[67]
LncRNA-H19	Cholangiocytes	HSC	--	Promoting HSC activation	--	[68]
LncRNA-H19	Cholangiocytes	Macrophage (Kupffer cells)	--	Promoting M1 polarization	--	[69]
LncRNA MT1DP	Liver	Kidney	--	Reinforcing nephrotoxicity of Cadmium.	--	[70]

VAT: visceral adipose tissue, BAT: brown adipose tissue, WAT: white adipose tissue, EC: endothelial cell, HSC: hepatic stellate cells, HPS: hepatopulmonary syndrome, ACSL: acyl-CoA synthetase long-chain, TCF7L2: transcription factor 7 like 2, PCK: phosphoenolpyruvate carboxykinase (PEPCK), PKC: protein kinase, TRAF3: tumour necrosis factor receptor (TNFR)-associated factor 3, FGF21: fibroblast growth factor 21, PPAR: peroxisome proliferator-activated receptor, IRS: insulin receptor substrate, BNDF: brain-derived neurotrophic factor, NADK: NAD^+^ kinase, KLF4: Kruppel-like factor 4, TRADD: TNFR type 1-associated death domain protein, SIRT1: the silent information regulator sirtuin 1, LAMC: lamin C, DKK1: Dickkopf-1, PTEN: phosphatase and tensin homolog, HO-1: heme oxygenase-1, AGPAT1: 1-acetylglycerol-3-phosphate O-acyltransferase 1, SHP: secondary hyperparathyroidism.

**Table 2:** Pathological RNAkines mediating the crosstalk between cancer cells and cells within the TME.

RNAkine	Stimuli	Donor	Recipient	Target	Effect	Mechanism	Ref.
miR-210	TIMP-1	LUAD	ECs (HUVECs)	FGFRL1, E2F3, VMP-1, RAD52, SDHD	Promoting angiogenesis	Activating PI3K/AKT signalling pathway.	[76]
miR-9	MYC OCT4	Glioma, medulloblastoma	ECs	S1P_1_, COL18A, THBS2, PTCH1, PHD3	Promoting angiogenesis and inflammation		[77, 78]
miR-25-3p	-	CRC	ECs	KLF2, KLF4	Promoting angiogenesis	Up-regulating the expression of VEGFR2, ZO-1, occluding and Caludin 5	[79]
miR-205	-	OvCa	ECs	PTEN	Promoting angiogenesis	Targeting PTEN-AKT pathway	[80]
miR-150	AGE	Monocytes	ECs Macrophage	c-Myb	Promoting angiogenesis	Regulating c-Myc in ECs or promoting VEGF secretion from M2.	[9, 81, 82]
miR-1247-3p	-	HCC	Fibroblast	B4GALT3	Promoting metastasis	Converting normal fibroblasts to cancer-associated fibroblasts by activating β1-integrin-NF-κB signalling	[85]
lncRNA LNMAT2	-	BCa	HLEC	PROX1	Lymphatic metastasis	Epigenetically upregulating PROX1 expression.	[86]
miR-92a-3p	-	HCC (Huh7 with selective pressure)	Epithelial cells	PTEN	Promoting EMT	Regulating Akt/Snail signalling.	[84]
miR-214	-	Various types of Cancer cells	T cells	PTEN	Treg expansion	Increasing population of CD4^+^CD25^high^Foxp3+ regulatory T cells (Treg) and mediating immune evasion.	[88]
circGSE1	-	HCC	T cells	miR-324-5p	Treg expansion	Silencing miR-324-5p/TGFBR1/Smad3 axis.	[89]
miR-25-3p miR-130b-3p miR-425-5p	CXCL12/CXCR4	CRC	Macrophage	PTEN	M2 polarisation	Activating PI3K/AKT signalling pathway.	[92]
miR-21-3p miR-125b-5p miR-181d-5p	Hypoxia	EOC	Macrophage	SOCS4 SOCS5	M2 polarisation	Increasing the phosphorylation of STAT3	[94]
miR-310a-3p	Hypoxia	PC	Macrophage	PTEN	M2 polarisation	Activating PTEN/PI3Kγ signalling pathway and promoting the secretion of Arginase, IL-10 and TGF-β	[94]
Circ0048117	Hypoxia	ESCC	Macrophage	miR-140	M2 polarisation	Activating TLR4 pathway and promoting the secretion of Arg1, IL-10 and TGF-β	[96]
miR-23a-3p	-	HCC	Macrophage	PD-L1	Inhibiting T cell function	Upregulating PD-L1 in macrophage	[90]
circRNA_002178	-	LUAD	T cells	miR-34	Inhibiting T cell function	Inducing PD-L1 expression via sponging miR-34	[91]
miR-122	-	BC	Stomal fibroblast	PKM	Metabolic reprogramming	Suppressing glucose uptake of non-tumour cells in premetastatic niche to facilitate disease progression	[97]
miR-105	MYC	BC	CAF	MYC	Metabolic reprogramming	Enhancing glucose and glutamine metabolism (nutrient replete) or converting metabolic waste into energy-rich metabolites (nutrient deprived)	[98]
miR-122	-	BC	Skeletal muscle	O-GlcNAc transferase	Promoting skeletal muscle proteolysis		[73]
miR-122	-	BC	Pancreas	PKM	Systematic glucose reprogramming	Impairing insulin secretion and systemic glucose homeostasis.	[74]

TIMP-1: Tissue inhibitor of metalloproteinases-1, EC: endothelial cells, HLEC, human lymphatic endothelial cells, LUAD: Lung adenocarcinoma, CRC: Colorectal cancer, OvCa: ovarian cancer, HCC: hepatocellular carcinoma, miR-23a-3p: Epithelial ovarian cancer, PC: pancreatic cancer, ESCC: esophageal cell squamous carcinoma, BCa : bladder cancer, BC: Breast cancer.

## Box 1 The potential and challenge for circulating ncRNA as disease fingerprints

Circulating ncRNAs have an untapped potential as valuable biomarkers for disease diagnosis, prognosis, and prediction due to their stable presence in the bloodstream and association with various diseases [108]. Ideal biomarkers are specific, sensitive, non-invasive, stable, and reproducible. Circulating ncRNAs meet some of these criteria, such as stability in the bloodstream and non-invasive accessibility. However, specificity, sensitivity and reproducibility challenges still need to be addressed to establish circulating ncRNAs as reliable disease fingerprints.

Various studies have demonstrated differential expression of miRNAs, lncRNAs, circRNAs and tsRNAs in the plasma or serum of individuals with a wide range of diseases, including obesity [109], type 2 diabetes [109, 110], cardiovascular disease [111, 112], neurodegenerative disease [108, 113] and various types of cancer [108, 114, 115]. The sensitivity and specificity of some ncRNAs as disease biomarkers have been significantly strengthened. For example, the upregulation of miR-222 in obesity and overweight has been confirmed by three independent investigations [116–118], and the levels of miR-122 are increased in the plasma or serum of individuals with obesity and insulin resistance and decreased in those who underwent surgery-induced weight loss [119, 120]. Therefore, independent studies repeated on similar or related sample cohorts provide more substantial evidence of the association between circulating ncRNAs and diseases.

Despite the promising potential of circulating ncRNAs as biomarkers, several challenges persist. Most studies focus on circulating miRNAs as biomarkers, but inconsistencies in altered miRNA levels across studies raise concerns about their reproducibility. Establishing a standardised procedure for assessing circulating miRNAs is essential. Different methods and systems, such as qPCR with various probes, TaqMan low-density array, small RNA sequencing or microRNA sequencing, have been employed to measure circulating miRNA levels. Additionally, normalising quantitative data of circulating miRNA poses a significant challenge for cross-sample comparison due to the lack of internal control for circulating miRNA. Some studies use circulating miRNAs that do not change in a specific disease as endogenous controls, such as miR-16 and miR-106, utilised for metabolic disease [109]. Their applicability is limited since they have not yet been shown to remain unaltered across various types of disease. Another approach involves spiking in an exogenous miRNA sequence (e.g., *Caenorhabditis elegans* miR-39) as an external control, which fails to reflect individual-specific variations [121]. Therefore, establishing a standardised assessment process, including RNA isolation, quality control, and determination and quantification methods, is imperative to ensure the reproducibility of circulating miRNA detection.

## Box 2 Selective and regulated secretion of extracellular ncRNA

The secretion of extracellular ncRNAs is a finely regulated process rather than a result of a passive cellular leakage. This notion is supported by the substantial variations observed in the spectra of extracellular RNAs, including miRNAs, lncRNAs, and circRNAs across diverse physiological and pathological conditions [6, 7, 109, 114]. Although the precise mechanisms behind ncRNA loading and packaging into EVs under specific conditions remain unclear, one promising mechanism involves sequence motif-determined sorting of EV ncRNAs. These motifs, responsible for ncRNA secretion, are termed EXOmotif, while those responsible for ncRNA retention are termed CELLmotif or CLmotif [13, 122]. Sequence motifs are recognised by specific RNA-binding proteins (RBPs) that act as the machinery for localising ncRNAs into EVs. Heterogeneous nuclear ribonucleoproteins (hnRNPs), a family of RBPs, have been identified as crucial players in ncRNA sorting. For instance, SUMOylated hnRNPA2B1 recognises motif GGAG, localising specific ncRNAs into EVs, such as miR-198 [122]. In addition, HNRNPA1 and HnRNPA2B1 can recognise the lncRNAs *ELNAT1* and *LNMAT2*, mediating their packaging into EVs [123]. Notably, the expression of many RBPs is regulated by pathological states. Increases in hnRNPA2B1 expression have been observed in lung, pancreatic adenocarcinoma, and breast cancer cells [106]. Therefore, unravelling the regulatory axis responsible for disease-related RBP-ncRNA secretion may shed light on how the secretion of ncRNAs can accurately respond to specific biological stimuli. This mechanism may explain why the secretion of ncRNAs follows a stimulus-response pattern similar to that of conventional hormones or cytokines.

## Glossary

### Abbreviations

Alcoholic Hepatitis (AH): alcohol-associated liver disease characterized by acute liver inflammation caused by heavy alcohol use.
Circular RNA (circRNA): RNA transcript that forms a close loop with both ends covalently connected. The structure makes circRNA resistant to exonuclease. CircRNA influences gene expression via diverse ways: including competing miRNAs as a sponge, regulating mRNA transcription, splicing and stability.
Chemokine: small signalling molecules that regulate immune responses by acting as chemoattractant to guide the migration of cells. They are small proteins or peptides that comprise four main subfamilies: CXC, CC, CX_3_X, and XC. They accomplish the function by binding with G protein-coupled receptors (GPCR).
Cytokines: proteins or peptides made by certain immune cells or non-immune cells (endothelial cells and fibroblasts) and affect the immune system. Examples are interferons (IFNs), interleukins (ILs), lymphokines and tumour necrosis factors (TNFs). They exert a wide range of immunoregulatory actions by interacting with cytokine receptors, activating signal transduction.
DICER: a crucial enzyme for miRNA biogenesis, which recognizes and cleaves the pre-miRNA loop hairpin, generating miRNA duplex with a length of ∼ 22-nt.
Epithelial-mesenchymal-transition (EMT): a process known as trans-differentiation of epithelial cells into mesenchymal cells that is involved in development, wound healing, fibrosis and cancer progression.
EV/exosomal miRNA: miRNA that are transferred via EV/exosome.
Exosome: main subpopulation of EV with a diameter ranging from 30-150 nm. Exosomes are originated intracellularly from endocytic pathway. The inward budding of endosomal membrane yields small vesicular structures within the endosome lumen to form multivesicular bodies (MVB). Exosomes are released extracellularly when MVB fuse with plasma membrane.
Extracellular RNA: RNA presented in extracellular space, including in various body fluids (e.g., serum plasma, saliva, urine and milk). They are presented by enclosed in EVs, or associated with lipoproteins (e.g., HDL) and RNA-binding proteins (e.g., AGO2). Many types of RNA species are found presenting in extracellular space, such as mRNA, miRNA, lncRNA, rRNA, tRNA, tsRNA, snoRNA, snRNA, mitochondrial RNAs, and artificially designed siRNA or guide RNA (such as Cas9-associated RNA).
Extracellular vesicle (EV): Heterogeneous group of membrane-bound particles naturally released by cells into extracellular space. EV contains various types of cytosolic material, including miRNA, mRNA, lipids, and proteins.
Growth factor: cytokines or hormones that regulate cell differentiation and proliferation. Examples include epidermal growth factor (EGF), fibroblast growth factor (FGF), and vascular endothelial growth factor (VEFG).
Hormone: signalling molecules that are produced by glands throughout the body, including eicosanoids, steroids, amino acid derivatives, protein/peptides, and gases. They are released in response to specific stimuli into circulation, and act in paracrine, endocrine or autocrine manner. The signalling types of hormones include endocrine, paracrine, autocrine and intracrine. They regulate fundamental physiological functions, including growth and development, metabolism, and reproduction.
Long noncoding RNA (lncRNA): a class of > 200-nt-long RNA transcripts that are not encoding proteins. LncRNA can regulate gene expression by multiples ways, including acting sponge of a miRNAs, regulating epigenetic modifications, protein/RNA stability, and translation.
MicroRNA (miRNA): small noncoding regulatory RNAs (19-22 nucleotides) that post-transcriptionally regulate gene expression by binding to specific mRNAs, leading to either mRNA degradation or inhibition of mRNA translation.
Microvesicle: main subpopulation of EV with a diameter ranging from 50-1000 nm. They are derived from outward budding of the plasma membrane.
Myoblast: the mononucleated quiescent satellite cells that defined as the precursor of muscle cells.
Myogenesis: the formation of skeletal muscular tissues.
Myotube: a long liner multinucleated fibre formed through the fusion of developmental myoblasts.
Secreted RNA: is a broad term used to describe a class of RNA molecules that are actively released by cells into the extracellular space.
Small nucleolar RNA (snoRNA): a family of conserved noncoding RNAs that have essential roles in guiding the chemical modifications and processing of other RNA, such as rRNA.
Tumour microenvironment (TME): the environment located in the vicinity of the tumour that are composed of multiple stroma cells, such as immune cells, fibroblasts, and endothelial cells, as well as noncellular components, such as extracellular matrix (ECM) and signalling molecules.
tRNA-derived small RNA (tsRNA): noncoding RNAs with 18-40 nucleotides in length that are derived from precursor or mature tRNAs.
